# Spermatogonial stem cell transplantation into nonablated mouse recipient testes

**DOI:** 10.1016/j.stemcr.2021.05.013

**Published:** 2021-06-17

**Authors:** Hiroko Morimoto, Narumi Ogonuki, Mito Kanatsu-Shinohara, Shogo Matoba, Atsuo Ogura, Takashi Shinohara

**Affiliations:** 1 Department of Molecular Genetics, Graduate School of Medicine, Kyoto University, Yoshida Konoe, Sakyo-ku, Kyoto 606-8501, Japan; 2 RIKEN, BioResource Research Center, Tsukuba 305-0074, Japan; 3 AMED-CREST, AMED, 1-7-1 Otemachi, Chiyodaku, Tokyo 100-0004, Japan

**Keywords:** blood-testis barrier, homing, GDNF, niche, spermatogonial stem cell

## Abstract

Spermatogonial transplantation has been used as a standard assay for spermatogonial stem cells (SSCs). After transplantation into the seminiferous tubules, SSCs transmigrate through the blood-testis barrier (BTB) between Sertoli cells and settle in a niche. Unlike in the repair of other self-renewing systems, SSC transplantation is generally performed after complete destruction of endogenous spermatogenesis. Here, we examined the impacts of recipient conditioning on SSC homing. Germ cell ablation downregulated the expression of glial cell line-derived neurotrophic factor, which has been shown to attract SSCs to niches, implying that nonablated niches would attract SSCs more efficiently. As expected, SSCs colonized nonablated testes when transplanted into recipients with the same genetic background. Moreover, although spermatogenesis was arrested at the spermatocyte stage in *Cldn11*-deficient mice without a BTB, transplantation not only enhanced donor colonization but also restored normal spermatogenesis. The results show promise for the development of a new transplantation strategy to overcome male infertility.

## Highlights

• Spermatogonial stem cells (SSCs) can engraft in nonablated testes
• GFRA1^+^ spermatogonia are relatively resistant to busulfan
• GDNF is downregulated after germ cell deficiency
• SSC transplantation into Cldn11-deficient testes restores spermatogenesis

## Graphical abstract

**Figure:**
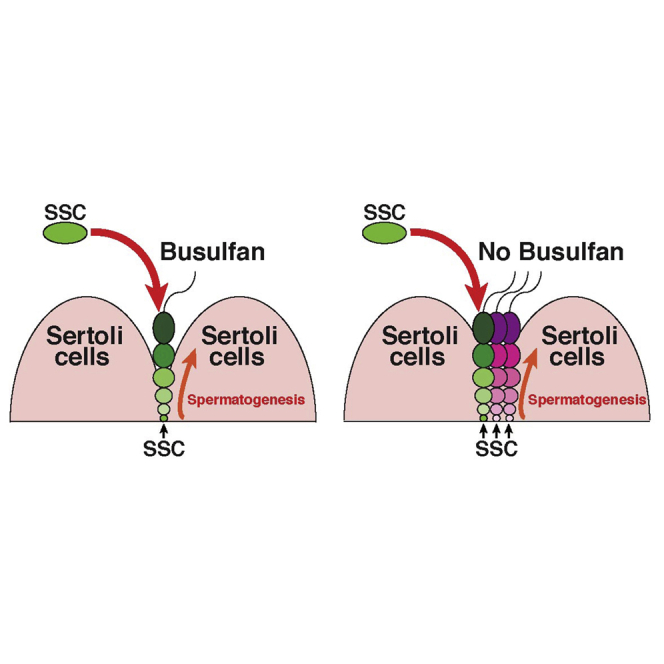

## Teaser

In this article, Shinohara and colleagues show that spermatogonial stem cells can colonize and produce offspring in nonablated recipients.

## Introduction

A spermatogonial transplantation technique was developed in 1994. With this technique, donor spermatogonial stem cells (SSCs) were observed to migrate into niches in recipient mice (Brinster and Zimmermann, 1994). Transplanted SSCs proliferated to make chains or networks of spermatogonia on the basement membrane within 2 weeks after transplantation (Nagano et al., 1999). As donor cell colonies became larger, differentiating germ cells appeared in the center of each colony, and sperm were finally found at 2–3 months after transplantation. With the transplantation of a sufficient number of SSCs, offspring can be born from the donor cells by mating the recipient males with wild-type females (Brinster and Avarbock, 1994). The most striking observation of the spermatogonial transplantation experiment was the passage of SSCs through the blood-testis barrier (BTB). Because the BTB divides each seminiferous tubule into the adluminal and basal compartments, transplanted SSCs must migrate through the BTB from the adluminal compartment into the basal compartment before reaching the niche on the basement membrane. However, normal spermatogenesis progresses from the basal compartment to the adluminal compartment; thus, SSCs were not expected to undergo this physiologically unusual migration. The experimental findings indicate that SSCs exhibit a unique migratory activity toward niches and that spermatogenesis can be reconstituted via self-renewing division. Because SSCs are the only cell type that can produce this result, spermatogonial transplantation has been used as a standard functional assay of SSCs, and it is expected that the technique will be used for the treatment of male infertility (Kubota and Brinster, 2018).

Compared with other tissues, one of the distinct features of SSC transplantation experiments is the timing of transplantation. In general, donor stem cells are transplanted immediately after the depletion of endogenous stem cells. For example, in hematopoietic stem cell (HSC) transplantation, recipients are irradiated to remove endogenous HSCs, and donor bone marrow cells are transplanted within a short period of time, usually on the same day after irradiation (Till and McCulloch, 1961). If the irradiated mice did not undergo transplantation, they would die due to bone marrow failure within 2–3 weeks. Therefore, HSCs need to be transplanted soon after irradiation. However, a loss of SSCs does not compromise the health of the recipient. Moreover, because transplantation into empty testes allows more donor cells to be transplanted easily, spermatogonial transplantation has been traditionally carried out using recipients with completely empty tubules.

For spermatogonial transplantation, recipients are generally prepared by treating the animals with busulfan, a chemical reagent that specifically removes a significant proportion of the endogenous SSC (Bucci and Meistrich, 1987; Jackson et al., 1962). Because busulfan preferentially kills primitive spermatogonia, it usually takes more than one cycle of spermatogenesis (35 days in mice) to create empty seminiferous tubules. Based on its effectiveness to remove SSCs, almost all of the published spermatogonial transplantation studies are based on busulfan-treated animals. SSCs can also colonize the seminiferous tubules of congenitally infertile mutant mice, such as WBB6F1-W/W^v^ (W) mice that lack nearly all endogenous germ cells from the time of birth (Brinster and Zimmermann, 1994). In animals where Sertoli cells have not been exposed to germ cells, mature Sertoli cells can still support donor SSC-derived spermatogenesis and offspring production (Brinster and Avarbock, 1994). Both models are used widely for spermatogonial transplantation experiments and exhibit comparable SSC colonization efficiency (Kanatsu-Shinohara et al., 2016a).

Although spermatogonial transplantation is conceptually similar to HSC transplantation, it has remained unknown whether the creation of empty niches and/or lack of many layers of endogenous germ cells are critical for successful SSC homing. In the present study, we examined the impacts of host factors on SSC homing. We evaluated the efficiency of donor cell colonization by focusing on the amount of endogenous germ cells and presence of the BTB. We also evaluated the extent of damage to the microenvironment from busulfan treatment. Our results showed that it was not necessary to completely remove endogenous germ cells for SSC transplantation and that disruption of the BTB allowed extensive donor cell colonization despite the presence of endogenous SSCs. Thus, our results raise a new possibility to use nonablated recipients for spermatogonial transplantation, which has important implications in future clinical application.

## Results

### Evaluation of spermatogenesis recovery after busulfan treatment

**Figure 1:**
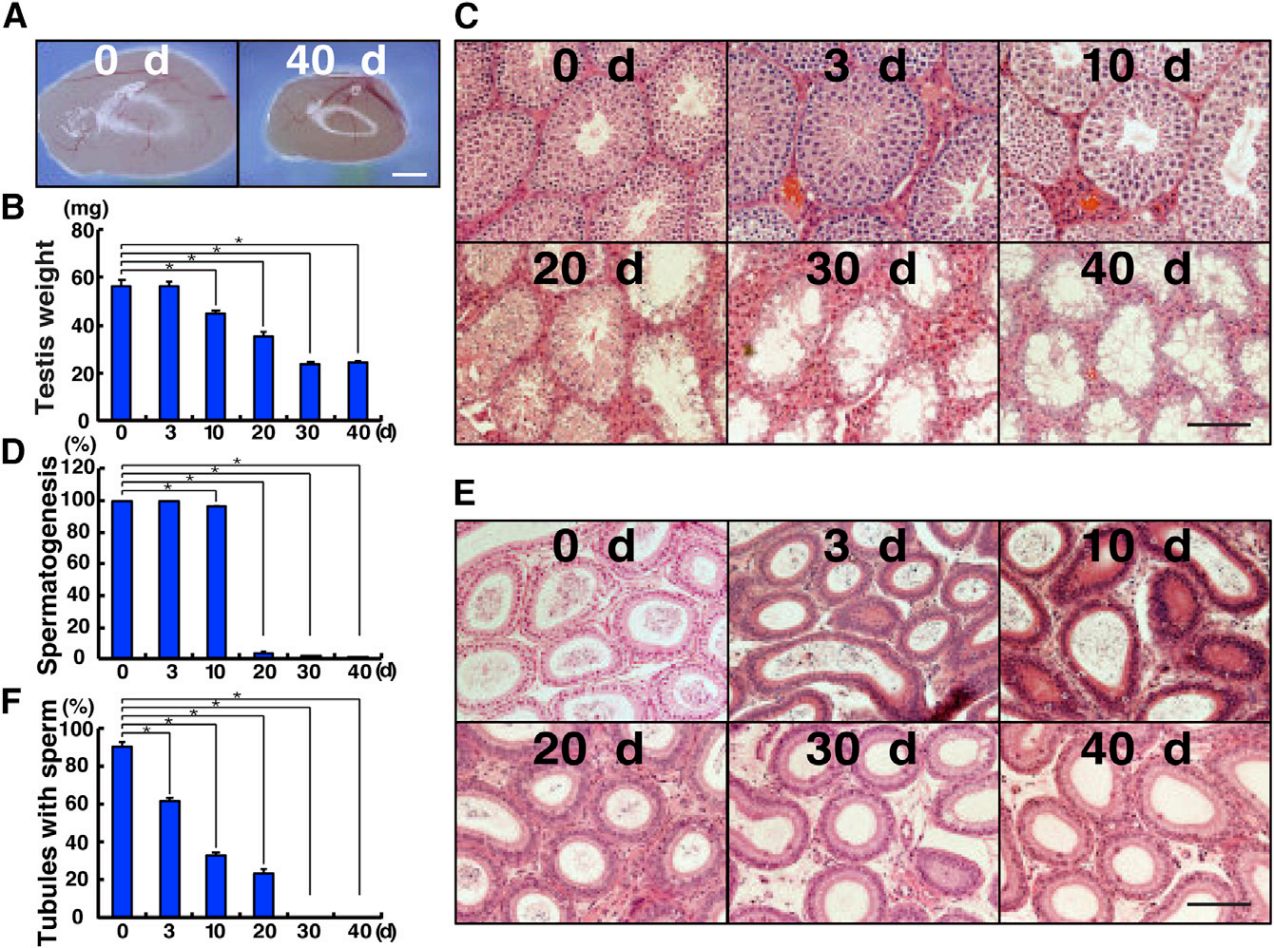
Evaluation of spermatogenesis levels after busulfan treatment (A) Macroscopic appearance of busulfan-treated testis. Busulfan treatment reduces the size of the testis. (B) Testis weights (n = 6–10 testes). Testis weight was significantly reduced after 10 days when compared with untreated testes. (C) Histological appearance of busulfan-treated testis. More empty tubules were found at later time points. (D) Number of seminiferous tubules in which spermatogenesis was observed (n = 5–8 testes). (E) Histological appearance of busulfan-treated epididymis. Sperm were gradually lost after busulfan treatment. (F) Number of the epididymal tubules in which spermatozoa were observed (n = 3 epididymides). Stain: hematoxylin and eosin (H&E) (C and E). Asterisk indicates statistical difference (p < 0.05). The numbers indicate days (d) after busulfan. Scale bars, 1 mm (A) and 50 μm (C and E).

We first evaluated the regeneration of spermatogenesis in 4-week-old wild-type mice after busulfan treatment. After busulfan was injected intraperitoneally (44 mg/kg), the testes of treated animals were recovered at 3, 10, 20, 30, or 40 days (Figure 1A). Because one cycle of spermatogenesis is approximately 35 days in mice, the total experimental period covered more than one cycle of spermatogenesis. At each sampling time point, testicular weight was recorded before histological analyses.

Testis weight decreased in a stepwise manner after busulfan treatment (Figure 1B). No significant difference was observed on day 3 after busulfan treatment, but the difference was significant on day 10 after treatment. Testis weight decreased even more on days 20 and 30 after treatment. On day 30, testis weight decreased to 41.7% of that of the untreated testes (23.6 versus 56.6 mg). Testis weight did not appear to increase after 40 days, implying that almost all the germ cells had disappeared by day 30 after busulfan treatment.

To confirm these observations, we conducted histological analysis of the busulfan-treated testes (Figure 1C). No apparent changes were observed on days 3 and 10 after busulfan treatment, and almost all seminiferous tubules contained germ cells (Figure 1D). Epididymal spermatozoa were also observed at these time points (Figures 1E and F). However, abnormal spermatogenesis was evident on day 20. Although numerous elongated spermatids were observed, spermatogonia and spermatocytes were rarely noted in treated testes. Moreover, most of the space within the seminiferous tubules was empty. Nevertheless, epididymal spermatozoa were still present at this point. On day 30 after busulfan treatment, virtually all seminiferous tubules lacked germ cells, and no spermatozoa were found in their epididymides. Similar histological observations of the testes were made on day 40 after busulfan treatment. These results confirm that the diploid spermatogonia stage is sensitive to busulfan treatment (Bucci and Meistrich, 1987).

### Immunostaining of busulfan-treated testes using germ cell markers

To quantify the busulfan-induced damage to germ cells, we performed immunostaining of busulfan-treated testes using antibodies for GFRA1, a component of the glial cell line-derived neurotrophic factor (GDNF) receptor and a marker for A_single_ (A_s_), A_paired_ (A_pr_), and some A_aligned_ (A_al_) undifferentiated spermatogonia; CDH1, a marker for all undifferentiated spermatogonia; KIT, a marker for differentiating spermatogonia; and SYCP3, a marker for spermatocyte. We also performed lectin-immunostaining using peanut agglutinin (PNA) to evaluate the number of haploid cells. For the analyses using spermatogonia markers, the number of individual spermatogonia in the seminiferous tubules was counted. For SYCP3 and PNA staining, the number of tubules with a positive stain was counted because there were too many cells in each tubule to count individually.

**Figure 2:**
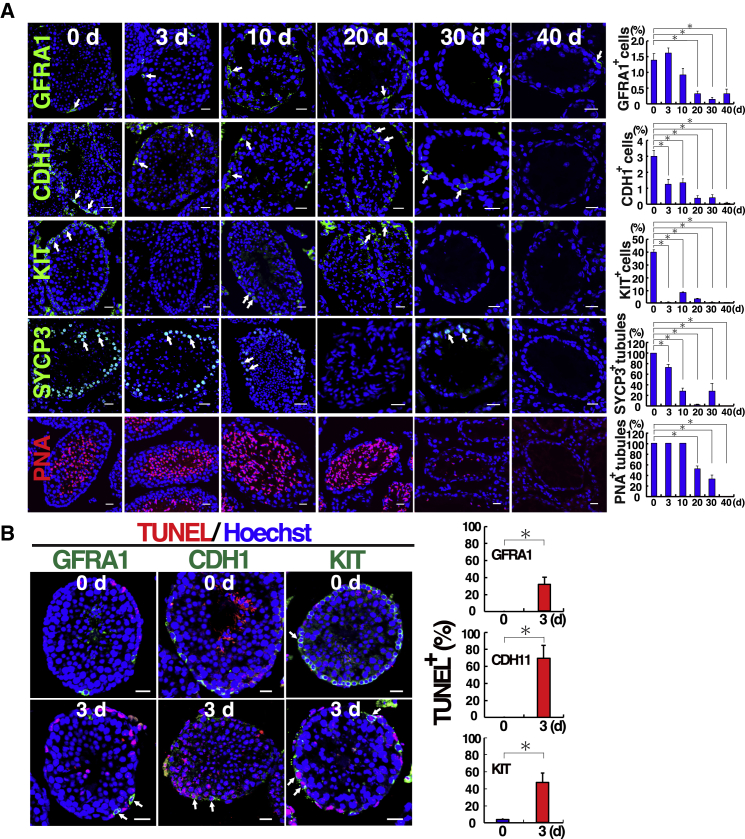
Immunostaining of busulfan-treated testes (A) Immunostaining of busulfan-treated testes with spermatogonia (GFRA1, CDH1, and KIT), spermatocyte (SYCP3), and haploid cell (PNA) markers. Spermatogonia and seminiferous tubules staining positive for each marker were quantified (n = 13–34 tubules for GFRA1, 9–24 tubules for CDH1, 13–40 tubules for KIT; n = 9–20 tubules for SYCP3; n = 16–20 tubules for PNA). Arrows indicate cells and tubules expressing the markers. For GFRA1, CDH1, and KIT, the numbers of spermatogonia in the tubule were counted. Because numerous germ cells were found by SYCP3 or PNA staining, tubule numbers were counted for SYCP3 and PNA. Note the persistent staining of GFRA1 throughout the experimental period. (B) Immunohistochemical analysis of apoptotic GFRA1^+^, CDH1^+^, and KIT^+^ cells using TUNEL staining. The spermatogonia with individual markers were quantified 0 and 3 days after busulfan (n = 10–19 tubules for GFRA1, 5–11 tubules for CDH1, 10–12 tubules for KIT). Arrows indicate cells expressing both TUNEL signals and differentiation markers. All types of spermatogonia showed increased apoptosis after busulfan. Stain: Hoechst 33342 (A and B). Asterisk indicates statistical difference (p < 0.05). The numbers indicate days (d) after busulfan. Scale bars, 20 μm (A and B). See also Table S1.

Based on spermatogonia counts, the number of GFRA1^+^ undifferentiated spermatogonia did not change significantly on day 3 after busulfan treatment (Figure 2A). By contrast, significantly fewer CDH1^+^ undifferentiated spermatogonia and KIT^+^ differentiating spermatogonia were observed at the same time point. In particular, the most drastic change was observed with the KIT marker, as virtually all KIT^+^ spermatogonia were lost at this point. Although the number of SYCP3^+^ tubules decreased by approximately 25%, all seminiferous tubules contained numerous PNA^+^ cells.

On day 10 after busulfan treatment, the number of GFRA1^+^ spermatogonia decreased compared with the untreated control, but the difference was not significant. The number of CDH1^+^ spermatogonia did not change significantly from that observed on day 3. However, the number of KIT^+^ spermatogonia increased slightly at this point. The number of SYCP3^+^ spermatocytes decreased further, to approximately 30% of the cell numbers in control samples, likely because of the rapid destruction of KIT^+^ spermatogonia. All seminiferous tubules still contained PNA^+^ haploid cells, although there appeared to be fewer spermatocytes in these tubules than in the control tubules.

On day 20 after busulfan treatment, the number of GFRA1^+^ spermatogonia decreased significantly to approximately ~21.4% of the original population size. Numbers of CDH1^+^ and KIT^+^ spermatogonia remained very low, indicating that these cells were almost completely destroyed. Consistent with this observation, very few tubules contained SYCP3^+^ spermatocytes. However, a significant proportion of the seminiferous tubules still contained PNA^+^ haploid cells. Consistent with the drastic loss of precursor cells, abnormalities in PNA expression pattern were noted by immunostaining of the testes.

On days 30 and 40 after busulfan treatment, the number of GFRA1^+^ spermatogonia decreased significantly. However, a small number of GFRA1^+^ cells were still observed in some samples, suggesting that spermatogenesis might regenerate in the long term. By contrast, CDH1^+^ and KIT^+^ spermatogonia were almost completely destroyed. However, the number of SYCP3^+^ spermatocytes increased transiently on day 30. This increase might have reflected the relatively large peak in KIT^+^ spermatogonia observed on day 10 after busulfan treatment. However, no SYCP3^+^ spermatocytes were found on day 40. The abundance of PNA^+^ haploid cells decreased significantly on day 30 after busulfan treatment, and no tubules contained PNA^+^ haploid cells on day 40. These results suggest that GFRA1^+^ spermatogonia comprise the most resistant population among the germ cell types analyzed.

### Apoptosis of spermatogonia after busulfan treatment

Because the immunostaining results indicated that GFRA1^+^ spermatogonia are relatively resistant to busulfan treatment, we used terminal deoxynucleotidyl transferase dUTP nick end labeling (TUNEL) staining to examine the levels of spermatogonia apoptosis more closely (Figure 2B). We compared busulfan-treated testes and untreated control testes on day 3 after busulfan treatment because most of the SSCs would have been destroyed at this point (Kanatsu-Shinohara et al., 2003a). Examination of stained control testes revealed that a relatively small number of surviving KIT^+^ spermatogonia exhibited TUNEL^+^ signals. For the treated testes, however, busulfan treatment increased the numbers of TUNEL^+^ cells for not only KIT^+^ spermatogonia but also GFRA1^+^ and CDH1^+^ spermatogonia. Approximately 40% of the GFRA1^+^ and KIT^+^ spermatogonia underwent apoptosis, whereas approximately 70% of the CDH1^+^ spermatogonia exhibited a positive TUNEL stain. Although simple immunostaining indicated that the number of GFRA1^+^ cells was relatively similar to those of CDH1^+^ or KIT^+^ spermatogonia (Figure 2A), these results imply that GFRA1^+^cells were also significantly damaged on day 3 after busulfan treatment.

### Loss of GDNF expression after germ cell depletion

**Figure 3:**
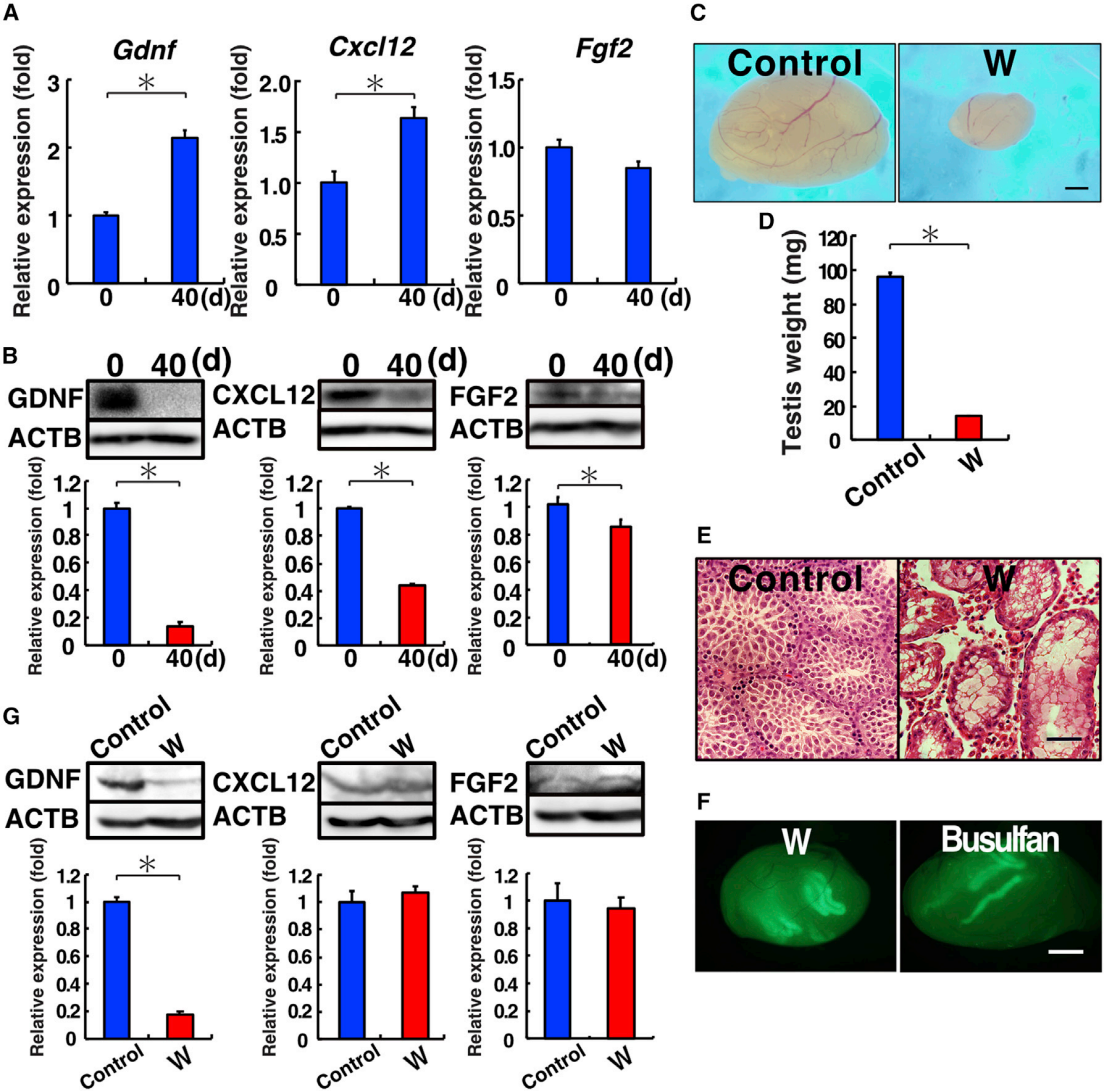
Impacts of germ cells on the cytokine environment (A) Real-time PCR analysis of busulfan-treated testes (n = 4 testes). Both *Gdnf* and *Cxcl12* showed significantly increased expression, but no changes were found for *Fgf2*. (B) Western blot analysis of busulfan-treated testes (n = 3 samples). All types of cytokines showed significant downregulation after busulfan. The numbers indicate days (d) after busulfan. (C and D) Macroscopic appearance (C) and testis weights (D, n = 4 testes) of W testis. (E) Histological appearance of W testis showing complete lack of spermatogenesis. (F) Macroscopic appearance of W recipient testis transplanted with green mouse testis cells. Both types of recipient testes can support donor-derived spermatogenesis. (G) Western blot analysis of W testes (n = 3 samples). Only GDNF is downregulated in W testes. Stain: H&E (E). Asterisk indicates statistical difference (p < 0.05). Scale bars, 1 mm (C and F) and 50 μm (E). See also Tables S1 and S2.

We next evaluated the impacts of busulfan on the SSC microenvironment. Real-time PCR analysis was performed, and the expression of critical cytokines involved in self-renewal and homing, i.e., *Gdnf*, *Fgf2*, and *Cxcl12*, was assessed. Testes of untreated and busulfan-treated mice were sampled on day 40 after busulfan treatment. Based on mRNA levels, *Gdnf* and *Cxcl12* expression in treated testes increased 2.1- and 1.6-fold, respectively (Figure 3A). Conversely, *Fgf2* mRNA levels did not differ significantly between the treated and control testes. To confirm the results at the protein level, we next carried out western blot analysis. Based on signal intensity, busulfan treatment significantly decreased the expression of all three cytokines (Figure 3B). In particular, GDNF protein expression decreased the most, to 13.8% of that in untreated mice. CXCL12 (C-X-C motif chemokine ligand 12) and FGF2 (fibroblast growth factor 2) protein levels were significantly downregulated, albeit to lesser degrees.

Although these results suggest that the SSC microenvironment was significantly damaged by busulfan, these changes could also have been caused by the removal of germ cells. To distinguish between these possibilities, we examined the expression levels of the same cytokines using W mice that only had a small number of undifferentiated spermatogonia. Unlike busulfan-treated mice, which could undergo normal spermatogenesis before treatment, W mice congenitally lack spermatogenesis. The size of the W testis was significantly smaller than that of the wild-type control testis (Figures 3C and 3D), and no spermatogenesis was observed in histological sections of W testes (Figure 3E). Despite their smaller testis size, W mice can serve as recipients for spermatogonial transplantation experiments (Figure 3F). Because Sertoli cells in W mice are not damaged by busulfan treatment, the model could be used to deduce whether the downregulation of GDNF, CXCL12, and FGF2 was caused by busulfan-induced damage. Wild-type mice exhibiting normal KIT function were used as the control. Western blot analyses showed that GDNF was significantly downregulated in W testes (Figure 3G). However, CXCL12 and FGF2 expression did not differ significantly from that in the control. These results strongly suggest that GDNF downregulation in both busulfan-treated and W mice was due to a lack of germ cells.

### Spermatogonial transplantation into busulfan-treated testes

Because GDNF attracts SSCs (Dovere et al., 2013; Kanatsu-Shinohara et al., 2012), we hypothesized that SSCs may be able to colonize niches at earlier time points if GDNF is more strongly expressed. To test this hypothesis, we transplanted donor cells into the seminiferous tubules of busulfan-treated mice on days 3, 10, 20, 30, or 40 after the treatment. All mice with a [C57BL/6 (B6) × DBA2 F1] (BDF1) background were treated with busulfan at week 4 after birth and received the same number of donor C57BL6/Tg14(act-EGFP-OsbY01) (green) mouse testis cells (1 × 10^6^ cells per testis) with a B6 background. Donor cells were also transplanted into untreated wild-type mice as a control. At least four experiments were performed for each time point.

**Figure 4:**
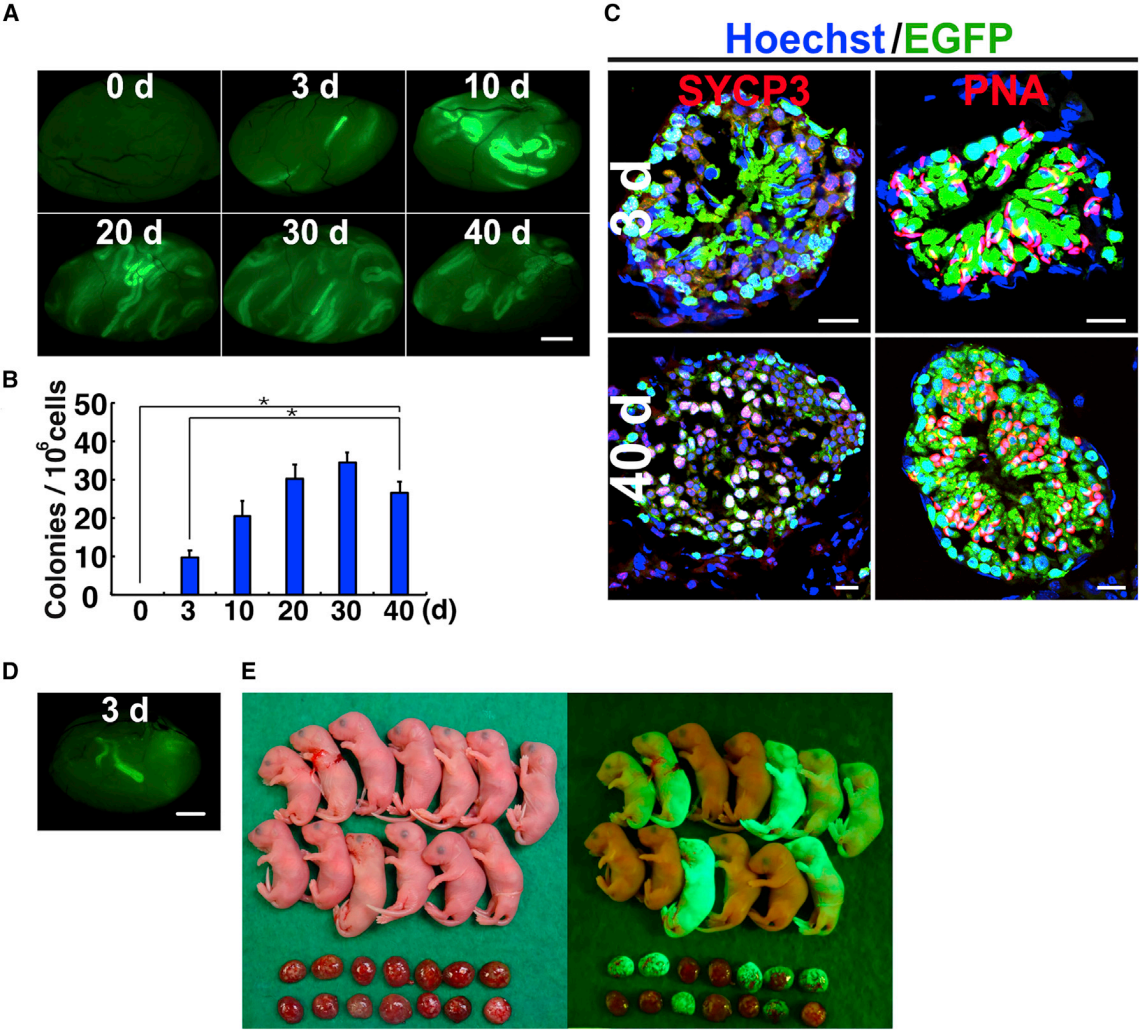
Functional analysis of busulfan-treated testes after spermatogonial transplantation (A) Macroscopic appearance of recipient testis 2 months after transplantation. Note the donor cell colonization in testis 3 days after busulfan. (B) Colony counts (n = 10–19 testes). Colonization was significantly better in testes 40 days after busulfan. (C) Immunostaining of recipient testis using spermatocyte (SYCP3) and haploid cell (PNA) markers. (D) Macroscopic appearance of recipient testis that was used for microinsemination. Donor cells were transplanted 3 days after busulfan. (E) Offspring born after microinsemination. Stain: Hoechst 33342. Asterisk indicates statistical difference (p < 0.05). The numbers indicate days (d) after busulfan. Scale bars, 1 mm (A and D) and 20 μm (C). See also Table S1.

The untreated wild-type testes were not colonized (Figure 4A). By contrast, donor cell colonization was evident in busulfan-treated recipients on day 3 after treatment (Figure 4B). Although the number of colonies was lower than those in other testes, 16 of 17 treated testes were colonized by donor cells, with a maximum of 25 colonies in one recipient. The number of colonies was significantly lower on day 3 after busulfan treatment than on day 40 (9.8 versus 26.5 colonies per 10^6^ transplanted cells). As expected, the number of colonies gradually increased as germ cells disappeared from the seminiferous tubules. Normal spermatogenesis was observed in immunostained recipient testes, as well as both SYCP3^+^ meiotic spermatocytes and PNA^+^ haploid cells (Figure 4C). These results suggest that the presence of endogenous spermatogenesis does not necessarily prevent donor cell colonization.

To test whether germ cells generated in these recipients are fertile, we used a microinsemination technique for offspring production. We collected testes from mice that received donor cells 3 days after busulfan treatment. Donor cell fluorescence was evident upon UV light (Figure 4D). The testes were refrigerated overnight and used for microinsemination on the next day. Germ cells in the tubules were dissected, and sperm from two testes were microinjected into oocytes by Piezo micromanipulator. In total, 76 embryos were produced, and 52 two-cell embryos were transferred into the oviducts of pseudopregnant mothers 24 h after sterile mating with vasectomized males. Cesarean section of the pseudopregnant mothers produced 25 progeny, 9 males, and 16 females (Figure 4E). Analysis of the offspring under UV light showed EGFP fluorescence in 2 males and 9 females, which confirmed the donor origin.

### Regeneration of spermatogenesis in B6 and BDF1 mice after busulfan treatment

From the preceding section, it appears that the presence of multiple layers of germ cells is not a major hurdle for colonization. This raises a possibility that SSCs might be transplanted into nonablated recipients. Such nonablative SSC transplantation would be beneficial for human patients who may want to undergo SSC transplantation to restore fertility. However, colonization did not occur in untreated testes, and the colonization efficiency is better in recipients with complete germ cell removal. Nevertheless, HSCs can colonize nonirradiated recipients by repeated injection of a large number of HSCs (Brecher et al., 1982). Moreover, SSCs on a BDF1 background proliferate more actively than those on a B6 background *in vitro* (Kanatsu-Shinohara et al., 2016b). Therefore, we reasoned that colonization in wild-type recipients might occur if both the donor and host mice have the same genetic background (B6).

**Figure 5:**
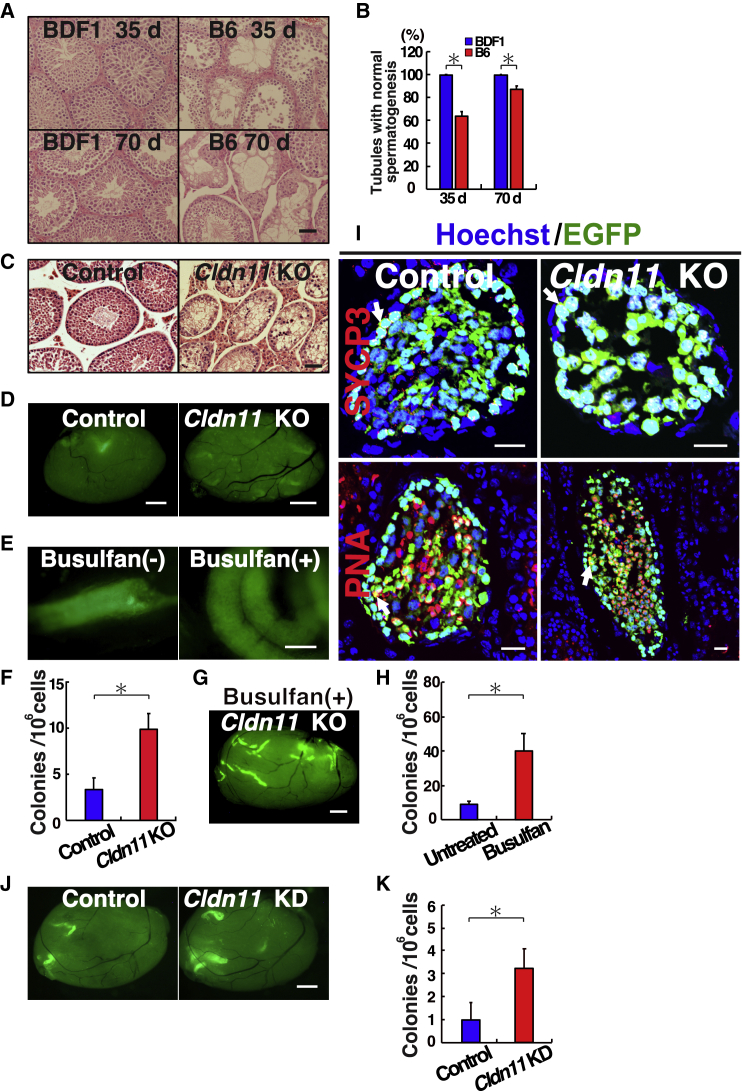
Functional analysis of *Cldn11* KO testes after spermatogonial transplantation (A) Histological appearance of BDF1 and B6 testes after busulfan treatment (15 mg/kg). Testes were collected at indicated time points. (B) Number of seminiferous tubules in which complete spermatogenesis was observed (n = 10 testes). (C) Histological appearance of *Cldn11* KO testis with defective spermatogenesis. (D) Macroscopic appearance of untreated recipient testis 2 months after transplantation. Colonization was enhanced in *Cldn11* KO testes. (E) Colony patterns in busulfan-treated (right) and untreated (left) wild-type recipient testes 2 months after transplantation. In control experiments using busulfan-treated mice, donor cells were transplanted 1 month after busulfan treatment. Colonies in untreated tubules were generally asymmetrical, while those in busulfan-treated tubules were symmetrical. (F) Colony counts in untreated testes showing enhanced colonization in *Cldn11* KO testes (n = 14 testes). (G) Macroscopic appearance of busulfan-treated *Cldn11* KO testis 2 months after transplantation. (H) Colony counts in busulfan-treated and untreated *Cldn11* KO testes showing enhanced colonization after busulfan (n = 13 testes for busulfan-treated, n = 14 testes for untreated). (I) Immunostaining of recipient testis using spermatocyte (SYCP3) and haploid cell (PNA) markers. Both SYCP3^+^ and PNA^+^ cells were found in *Cldn11* KO testes after transplantation. Arrows indicate cells with indicated markers. (J) Macroscopic appearance of recipient testis 2 months after *Cldn11* KD and donor cell transplantation. (K) Colony counts showing enhanced colonization after *Cldn11* KD (n = 12–13 testes). Stain: H&E (A and C), Hoechst 33342 (I). Scale bars, 50 μm (A), 20 μm (C and I), and 1 mm (D, E, G, and J). See also Tables S1 and S2.

We first compared the regenerative potential of SSCs in these strains *in vivo*. We administered the same dose of busulfan (15 mg/kg) into B6 or BDF1 mice and examined the degree of spermatogenesis regeneration at 35 days, which corresponds to one cycle of mouse spermatogenesis. We previously showed that busulfan at this dose can transiently disrupt spermatogenesis by partial depletion of SSCs on a B6 background (Kanatsu-Shinohara et al., 2003a). However, when the testes were examined after 35 days, all seminiferous tubules in BDF1 mice showed normal spermatogenesis (Figures 5A and 5B). In contrast, a significant number of seminiferous tubules showed abnormal spermatogenesis in B6 mice (Figures 5A and 5B). Many tubules contained partial spermatogenesis, and empty tubules were found. Spermatogenesis in BDF1 mice did not show any changes at 70 days after busulfan. Although regeneration was evident in B6 mice, tubules with abnormal spermatogenesis were still found (Figures 5A and 5B). These results suggested that SSCs in BDF1 mice have a stronger competitive advantage than those in B6 mice.

### Improved donor cell colonization in nonablated recipients in the absence of Cldn11

While these results raised a possibility that the genetic background may play a role in spermatogenesis regeneration and transplantation, we sought an additional factor that might improve colonization levels. One of the potential impediments for colonization is the BTB. The BTB has been considered to be a major barrier against successful SSC homing because transplantation into immature recipient testes before the BTB formation showed enhanced donor cell colonization (Shinohara et al., 2001). The inhibitory effect of the BTB on SSC colonization was also confirmed in a recent study using adult mice (Kanatsu-Shinohara et al., 2020). Therefore, modulation of the BTB might improve colonization efficiency, which can be possible in human patients by transient inhibition of the BTB proteins.

Based on these considerations, we designed an experiment in which B6 donor SSCs were transplanted into *Cldn11* knockout (KO) mice on a B6 background to test whether the lack of a BTB would lead to increased SSC colonization when transplantation was carried out in the same genetic background. CLDN11 is a major component of the BTB, thus *Cldn11* KO mice lack BTBs (Gow et al., 1999; Kitajiri et al., 2004). Spermatogenesis is severely compromised in these testes, and spermatogenic cells can only differentiate up to the preleptotene spermatocyte stage (Figure 5C). Because of the lack of a BTB and the reduced number of endogenous germ cells, we expected that donor SSCs might colonize more efficiently in *Cldn11* KO mice.

We transplanted testis cells from mature green mice into untreated wild-type and *Cldn11* KO mice. As a positive control for transplantation, we transplanted B6 testis cells into busulfan-treated wild-type and *Cldn11* KO recipients. Busulfan-treated mice were used at least 30 days after busulfan administration. Two months after transplantation, we sacrificed the recipient mice and analyzed their testes. Donor cell colonization occurred in all types of recipients (Figure 5D). However, upon close examination of recipient testes, colony morphology was heterogeneous in the untreated wild-type mice, which likely reflected the normal pattern of spermatogenic cell differentiation from SSCs. In busulfan-treated wild-type mice, colonies were generally longer and more symmetrical (Nagano et al., 1999), whereas those in wild-type recipients were shorter and often truncated (Figure 5E). In addition, we observed many colonies without apparent vertical differentiation, indicating that spermatogenesis was arrested before the formation of haploid cells.

Colonization occurred in 13 of 14 (92.9%) untreated *Cldn11* KO testes and 11 of 14 (78.6%) untreated wild-type testes. Enumeration of colony counts revealed that the number of colonies generated in *Cldn11* KO and wild-type control mice were 9.1 and 3.8 per 10^6^ transplanted cells, respectively (n = 14; Figure 5F), and the difference was significant. As expected, donor cell colonization occurred more extensively in busulfan-treated testes. The number of colonies generated in busulfan-treated and untreated *Cldn11* KO mice were 40.1 and 9.3 per 10^6^ transplanted cells, respectively (n = 13 for busulfan-treated *Cldn11* KO; n = 14 for untreated *Cldn11* KO) (Figures 5G and 5H). Interestingly, although haploid cells were never observed in untreated *Cldn11* KO testes before transplantation, immunostaining of untreated *Cldn11* KO recipient testes revealed the presence of not only SYCP3^+^ meiotic cells but also PNA^+^ haploid spermatids (Figure 5I), implying that spermatogonial transplantation rescued the spermatogenic defects caused by *Cldn11* deficiency.

To test whether endogenous *Cldn11* depletion in wild-type mice can improve colonization efficiency, we carried out *in vivo* knockdown (KD) of *Cldn11* using small interfering RNA (siRNA) prior to donor cell transplantation. Albeit at lower degrees compared with KO recipient mice, analysis of the recipients also enhanced donor cell colonization in wild-type mice (Figures 5J and 5K). Taken together, these results suggest that donor SSC colonization occurs even when endogenous spermatogenesis is not completely depleted.

## Discussion

One of the striking observations in this study was the decrease in levels of critical cytokines in busulfan-treated mice. In particular, GDNF is considered the most critical self-renewal factor because a decrease in GDNF levels has been shown to suppress spermatogenesis and cause infertility in *Gdnf* heterozygous KO mice (Meng et al., 2000). Although GDNF expression was thought to be limited to Sertoli cells, GDNF was more recently found to be also expressed in peritubular cells (Chen et al., 2016). The stage-specific cyclical distribution of GDNF along the basal surfaces of Sertoli cells has been visualized using whole-mount immunostaining (Johnston et al., 2011; Sato et al., 2011; Sharma and Braun, 2018; Tokue, et al., 2017). Double immunostaining of GDNF and GFRA1 also revealed the close localization of GDNF deposits and a subpopulation of GFRA1^+^ spermatogonia. In terms of *Gdnf* regulation, the removal of germ cells may increase GDNF expression because such treatment increases *Gdnf* mRNA, possibly through increased follicle-stimulating hormone secretion from the pituitary gland (Garcia et al., 2017; Ryu et al., 2006; Tadokoro et al., 2002; Zohni et al., 2012). JAG1 expressed by germ cells appears to suppress *Gdnf* expression via the NOTCH pathway in Sertoli cells (Garcia et al., 2017). Although these results are consistent with the results of our *Gdnf* mRNA analysis, our study implies that the regulation of GDNF translation is more complex as generally considered.

Because the Sertoli cells in busulfan-treated mice might have been damaged by the treatment, we analyzed W mice. Although the Sertoli cells in W mice had never been exposed to germ cells, GDNF levels in their testes decreased significantly, which contradicts its role as a conventional niche-derived factor. Therefore, the self-renewal and homing of SSCs in germ cell-depleted testes may be maintained by other cytokines. Indeed, although GFRA1 is considered an SSC marker, conflicting observations have been reported for GFRA1 expression in SSCs (Buageaw et al., 2005; Ebata et al., 2005). Furthermore, GDNF-independent self-renewal has been reported (Takashima et al., 2015). Consistent with our observations in mice, GDNF was similarly downregulated in human testes with Sertoli cell-only syndrome (Singh et al., 2017). In fact, GFRA1 expression is undetectable in a subpopulation of A_dark_ undifferentiated spermatogonia in humans (Caldeira-Brant et al., 2020). Perhaps a lack of germ cells may contribute to weak GDNF expression in such cases. These results prompt re-evaluation of the role of GDNF in SSC maintenance.

Unexpected kinetics of spermatogonia depletion was observed in our analysis of busulfan-treated mice. Previously, busulfan was shown to damage A_s_ and A_pr_ spermatogonia in a relatively selective manner (Bucci and Meistrich, 1987). Consistent with this study, SSC numbers decreased significantly on day 3 after busulfan treatment (Kanatsu-Shinohara et al., 2003a). Therefore, we expected that only GFRA1^+^ spermatogonia would be selectively eliminated in busulfan-treated testes. On the contrary, almost all KIT^+^ spermatogonia were destroyed more rapidly within 3 days, whereas GFRA1^+^ undifferentiated spermatogonia remained as long as 40 days. Although differentiating spermatogonia constitute the cell type most sensitive to the effects of antineoplastic agents (Lu and Meistrich, 1979; Meistrich, 1984; Parekh et al., 2019), it was thought that the A_s_ and A_pr_ spermatogonia were more sensitive for busulfan (Bucci and Meistrich, 1987). A critical difference between the previous study and the current study is the method of analysis; we used immunohistochemistry to detect the remaining cell types, while colony regeneration *in situ* was morphologically evaluated in the previous study. In addition, the mouse genetic background and busulfan dose should be considered. While C3H mice were administered with 40 mg/kg of busulfan in the previous study, BDF1 mice were treated with 44 mg/kg busulfan in the current study. Because our previous study showed efficient removal of spermatogenesis in C3H mice compared with mice in other strains (Kanatsu-Shinohara et al., 2010), A_s_ and A_pr_ spermatogonia are probably more sensitive to busulfan in a C3H background.

Another important observation was the efficient colonization of donor SSCs as early as 3 days after busulfan treatment. In all transplantation experiments, SSCs were able to colonize seminiferous tubules even before the complete removal of germ cells. On day 3 after busulfan, only KIT^+^ spermatogonia were significantly reduced in number and most of the other germ cells were still present in the recipient testes. However, the results of the transplantation experiment strongly suggest that vacant niches were already available at this time point. Therefore, most of the GFRA1^+^ spermatogonia were likely to be progenitors, and A_s_ spermatogonia were probably selectively damaged at this point, although this was not evident with simple GFRA1 immunostaining. Despite the decrease in GDNF, FGF2, and CXCL12 expression, the colonization efficiency was comparable between days 10 and 40. The fewer colonies observed on day 3 were likely due to damage from residual busulfan. Busulfan rapidly disappears within several hours from peripheral blood (Bouligand et al., 2007), but radiolabeled busulfan has been shown to persist for more than 72 h in rats (Meyers et al., 2017). Therefore, the transplanted SSCs might have been damaged from the remaining busulfan. Although busulfan-treated testes at this point still contained a significant number of germ cells, these germ cells did not hinder donor cell colonization. In this sense, SSC transplantation is similar to HSC transplantation in that stem cells can immediately colonize a niche even when the niche is surrounded by abundant progenitor cells.

Finally, we attempted to colonize the testes of untreated recipients. We did not expect to find colonies in untreated wild-type BDF1 recipients because we failed to observe any colonies in the preceding experiments. We also failed to observe colonization in our previous study using mice with mixed backgrounds (129/B6 background; Shinohara et al., 2002). However, colonization was observed in a significant proportion of B6 recipients. Therefore, genetic background is an important factor that influences SSC colonization efficiency. This was also suggested by the busulfan treatment of B6 and BDF1 mice, which showed enhanced regeneration of spermatogenesis in BDF1 mice. Moreover, in our experiments using *Cldn11* KO mice (B6 background), SSC colonization improved after the BTB was removed. Although the colonization efficiency was modest compared with that observed in busulfan-treated recipients, this can be increased by transplanting a larger number of SSCs because it is now possible to increase the number of SSCs *in vitro* (Kanatsu-Shinohara et al., 2003b). The increased colonization in *Cldn11* KO testes could be due to the lack of BTBs, a relatively weak competitiveness of endogenous B6 SSCs, or both. Interestingly, haploid cells developed in *Cldn11* KO mice. Intact *Cldn11* KO mice never form haploid cells, but we recently showed that autologous spermatogonial transplantation can restore spermatogenesis in *Cldn11* KO mice, implying that the BTB is dispensable for spermatogenesis (Kanatsu-Shinohara et al., 2020). In that experiment, *Cldn11* KO mice were treated with busulfan, which appeared to have reprogrammed CLDN3/5/11 expression in the Sertoli cells. However, our findings in this study imply that haploid cells can form even when endogenous germ cells are not removed. We speculate that nonphysiological SSC transmigration per se might have influenced tight junction protein (TJP) expression patterns and triggered haploid cell formation. Further studies are required to understand the relationship between TJPs and the suppression of spermatogenesis.

Our findings have several practical implications for the improvement of spermatogonial transplantation. First, transplantation can be performed without waiting for complete removal of endogenous germ cells, saving significant amounts of time. Although mouse spermatogenesis is relatively short (35 days), a complete cycle of spermatogenesis takes much longer in other species, such as 53 days in rats and 64 days in humans (Clermont, 1972). However, because busulfan excretion may take several days and the preparation and excretion of a large amount of busulfan is potentially dangerous, radiation appears to be practically safer and more useful. Moreover, localized irradiation is easier to perform on larger animals than in mice (Creemers et al., 2002). Second, it may be possible to transplant SSCs without disturbing endogenous hormones. In rats, the removal of germ cells causes severe edema and compromises the SSC microenvironment (Ogawa et al., 1999). However, early transplantation may avoid these problems because spermatogenesis can recover before edema formation. Finally, an optimized transient *in vivo Cldn11* KD protocol by siRNA will likely enhance donor cell colonization regardless of endogenous spermatogenesis. This is ideal because donor SSCs will be able to complete spermatogenesis when the BTB is restored.

Currently, there is a growing interest to apply a spermatogonial transplantation technique to restoring the fertility of prepubertal boys whose SSCs had been damaged or lost through chemo- or radiotherapy for cancers (Kubota and Brinster, 2018). Accordingly, transplantation experiments are now being developed in primate models (Hermann et al., 2012; Shetty et al., 2020). The busulfan or radiation treatment is given to the mice or other animals to model the young cancer survivors. According to the outcome of this study, it is now feasible that transplantation can be performed without complete removal of endogenous germ cells. However, there are at least two issues that require further studies. First is the relationship between endogenous germ cells and fertility restoration. It has been suggested that endogenous spermatogenesis promotes offspring production from donor cells (Brinster and Avarbock, 1994). Indeed, not all recipients can restore fertility even in mice by spermatogonial transplantation into completely empty seminiferous tubules (Kanatsu-Shinohara et al., 2016a). Another issue is the optimal timing of transplantation in young boys. Although immature recipients provide a better environment for donor cell colonization (Shinohara et al., 2001), they may not be hormonally ready for the full restoration of spermatogenesis if autologous transplantation is performed immediately after cytotoxic treatment. Investigation into these points using experimental animals will provide valuable information for the future development of infertility treatments based on spermatogonial transplantation.

## Experimental procedures

### Animals and microinjection procedure

Four-week-old wild-type and W mice with a WBB6F1 background were purchased from Japan SLC (Shizuoka, Japan). We also used 4-week-old BDF1 mice to investigate busulfan effects (Japan SLC). *Cldn11* KO mice were kindly provided by Dr. S. Tsukita (Osaka University, Suita, Japan). Busulfan was prepared as described previously by first dissolving the powder in dimethylsulfoxide at 8.0 mg/mL. An equal volume of sterile distilled water was then added to produce a final concentration of 4.0 mg/mL, and the solution was administered via intraperitoneal injection (Ogawa et al., 1997). For partial depletion of spermatogenesis, the solution was diluted with distilled water and injected into 4-week-old B6 and BDF1 mice (Japan SLC). Donor cells were prepared from green mice that were more than 8 weeks old (obtained from Dr. M. Okabe, Osaka University). Testis cells were prepared via a two-step enzymatic digestion procedure using type IV collagenase and trypsin (both from Sigma, St. Louis, MO), as described previously (Ogawa et al., 1997). For the microinjection of germ cells, dissociated single-cell suspensions (10^6^ cells/testis) were transplanted into seminiferous tubules via the efferent duct (Ogawa et al., 1997). Each injection filled 75%–85% of the seminiferous tubules. The Institutional Animal Care and Use Committee of Kyoto University approved all animal experimentation protocols.

### Statistical analyses

Results are presented as means ± SEM. Data were analyzed using Student's t tests. Multiple comparisons were performed using analysis of variance followed by Tukey's HSD test.

## Author contributions

H.M. carried out most of the experiments and analyzed data. N.O., S.M., and A.O. performed microinsemination. T.S. designed the experiment and carried out spermatogonial transplantation. M.K.-S. and T.S. wrote the paper.

## Supplemental information

Document S1. Supplemental experimental procedures and Tables S1 and S2

Document S2. Article plus supplemental information
