# Supplementary material for: Spermatogonial stem cell transplantation into nonablated mouse recipient testes
Source: Stem Cell Reports. 2021 Jun 17;16(7):1832–44. doi: 10.1016/j.stemcr.2021.05.013 (PMC8282430; doi:10.1016/j.stemcr.2021.05.013)
Supplement: Document S1. Supplemental experimental procedures and Tables S1 and S2 [file mmc1.pdf]

Stem Cell Reports, Volume *16*

Supplemental Information

Spermatogonial stem cell transplantation into nonablated mouse recipient testes

Hiroko Morimoto, Narumi Ogonuki, Mito Kanatsu-Shinohara, Shogo Matoba, Atsuo Ogura, and Takashi Shinohara

Supplementary Data for

Spermatogonial stem cell transplantation into nonablated mouse recipients

Hiroko Morimoto, Narumi Ogonuki, Mito Kanatsu-Shinohara, Shogo Matoba, Atsuo Ogura, and Takashi Shinohara

Supplemental Experimental Procedure

In vivo KD of Cldn11

To deplete *Cldn11* in vivo, 10 μl of siRNA against *Cldn11* (Silencer Select Pre-designed siRNA; Assay ID: s71159; Thermo Fischer, Waltham, MA) was vigorously mixed with 10 μl of invivofectamine 3.0 (Thermo Fisher). After incubation at 50 ° C for 30 min, an equal volume of phosphate-buffered saline (PBS) was added to the solution, which was then transferred onto ice before it was microinjected into the seminiferous tubules of wild-type B6 mice. Donor green testis cells (10^6 cells/testis) were transplanted on day 4 after in vivo KD.

Histological analysis

Samples were fixed in 4% paraformaldehyde for 2 h and embedded in paraffin blocks or OCT compound for sectioning. Paraffin sections were counterstained with hematoxylin and eosin. For immunostaining, samples were immersed in a blocking buffer (0.1%Tween 20, 3% bovine serum albumin, and 10% goat serum in PBS) for more than 1 h before incubation with the primary antibodies at 4 °C overnight. Next, the secondary antibodies were added, and the samples were incubated for 1 h at room temperature. The samples were counterstained with Hoechst 33342 (Sigma), and visualized using a confocal microscope (Fluoview FV1000D; Olympus, Tokyo, Japan). The antibodies used are listed in Table S1.

Terminal deoxynucleotidyl transferase dUTP nick end labeling (TUNEL) staining

Histological sections were stained with an In Situ Cell Death Detection Kit:TMR Red (Roche Applied Science, Indianapolis, IN) according to the manufacturer's instructions. Hoechst 33342 was used for counterstaining.

Real-time PCR

Total RNA was isolated using TRIzol reagent (Invitrogen, Carlsbad, CA). First-strand cDNA was produced using a Verso cDNA synthesis kit (Thermo Fisher). Real-time PCR was performed using StepOnePlus^{TM} real-time PCR system (Applied Biosystems, Cheshire, UK) and the *Power* SYBR Green PCR Master Mix (Applied Biosystems). Transcript levels were normalized according to *Hprt* expression. The PCR conditions were as follows: 95°C for 10 min, followed by 40 cycles of 95°C for 15 s and 60°C for 1 min. Each PCR experiment was performed in triplicate. PCR primers are listed in Table S2.

Western blot analysis

Samples were separated using SDS-PAGE and transferred to Hybond-P membranes (Amersham Biosciences, Buckinghamshire, UK). Membranes were then incubated with the primary

antibodies. Band intensity was measured using Image Gauge software (Fuji Film, Tokyo Japan). The antibodies used were listed in Table S1.

Colony counting

Recipient mice were sacrificed at 8 weeks post-transplantation. Donor cell colonies were counted under UV light. Donor cell clusters were defined as colonies when the entire basal surface of the tubule was occupied and the cluster measured at least 0.1 mm in length (Nagano et al., 1999).

Microinsemination

Recipient mice were sacrificed and their testes were dissected. Seminiferous tubules were dissociated by a fine stainless needle to release spermatogenic cells into PBS. Elongated spermatids or spermatozoa were microinjected into oocytes from BDF1 mice using a Piezo-driven micropipetter (PrimeTech, Ibaraki, Japan), as described previously (Ogonuki et al., 2006). Embryos at the 2-cell stage after 24 h in culture were transferred into the oviduct of the pseudopregnant ICR females. Offspring were born after cesarean section.

Supplemental References

Nagano, M., Avarbock, M. R., and Brinster, R. L. (1999). Pattern and kinetics of mouse donor spermatogonial stem cell colonization in recipient testes. Biol. Reprod. *60*, 1429-1436.

Ogonuki, N., Mochida, K., Miki, H., Inoue, K., Fray, M., Iwaki, T., Moriwaki, K., Obata, Y., Morozumi, K., Yanagimachi, R., and Ogura, A. (2006). Spermatozoa and spermatids retrieved from frozen reproductive organs or frozen whole bodies of male mice can produce normal offspring. Proc. Natl. Acad. Sci. USA *103*, 13098-13103.

Supplemental Table S1. Antibodies

**Antigen**	**Name**	**Company**
ACTB	Mouse anti-mouse ACTB	Sigma-Aldrich, St. Lois, MO
CDH1	Rat anti-mouse CDH1	Gift from Dr. M. Takeichi (RIKEN, CDB)
CXCL12	Rabbit anti-mouse CXCL12	Santa Cruz, Dallas, TX
FGF2	Goat anti-mouse FGF2	Santa Cruz, Dallas, TX
GDNF	Rabbit anti-mouse GDNF	Abcam, Cambridge, MA
GFRA1	Goat anti-rat GFRA1	R&D systems, Minneapolis, MN
HIF1A	Rabbit anti-human HIF1A	Santa Cruz, Dallas, TX
KIT	Rat anti-mouse KIT	eBiosicence, San Diego, CA
SYCP3	Rabbit anti-mouse SYCP3	NOVUS Biologicals, Littleton, CO
**Secondary reagents**		
Alexa Fluor 488 goat anti-rat IgG (H+L)		Molecular Probes, Carlsbad, CA
Alexa Fluor 488 donkey anti-goat IgG (H+L)		Molecular Probes, Carlsbad, CA
Alexa Fluor 555 donkey anti-rabbit IgG (H+L)		Molecular Probes, Carlsbad, CA
Alexa Fluor 568 goat anti-rabbit IgG (H+L)		Molecular Probes, Carlsbad, CA
HRP- donkey anti-goat IgG		Abcam, Cambridge, MA
HRP- goat anti-mouse IgG		Cell signaling, Danvers, MA
HRP- goat anti-rabbit IgG		Cell signaling, Danvers, MA
Rhodamine peanut agglutinin		Vector Laboratories (RL-1072)

Supplemental Table S2. PCR primers

**Genotyping**		
Cldn11	OSP5'-2: CTACTTCTCTAGGAGTGAGAC<br>OSP A: AGCACTGCTGCGACAGCGTGCA<br>intraNeo5: GCCTGCTCTTTACTGAAGGCTCTT<br>WT = 400 bp with OSP5'-2 and OSP A, KO = 600 bp with OSP 5'-2 and intraNeo5	
**qPCR**	**Forward**	**Reverse**
Cxcl12	GCTCTGCATCAGTGACG	CCAGGTACTCTTGGATCC
Fgf2	CTCTACTGCAAGAACGGCG	CATAGCAAGGTACCGGTTGG
Gdnf	GCCACTTGGAGTTAATGTCC	CTTCGAGAAGCCTCTTACCG
Hprt	GCTGGTGAAAAGGACCTCT	CACAGGACTAGAACACCTGC
